# SnO_2_ Quantum Dots Distributed along V_2_O_5_ Nanobelts for Utilization as a High-Capacity Storage Hybrid Material in Li-Ion Batteries

**DOI:** 10.3390/molecules26237262

**Published:** 2021-11-30

**Authors:** I. Neelakanta Reddy, Bhargav Akkinepally, Venkatesu Manjunath, Gaddam Neelima, Mogalahalli V. Reddy, Jaesool Shim

**Affiliations:** 1 School of Mechanical Engineering, Yeungnam University, Gyeongsan 38541, Korea; bhargav.aero@gmail.com; 2 Department of Physics, Sri Padmavati Mahila Visvavidyalayam, Tirupati 517502, India; drvmanju18@gmail.com; 3 Department of Physics, National Taiwan University, Taipei 10617, Taiwan; neelimareddy2011@gmail.com; 4 Nouveau Monde Graphite, Saint Michel Des Saints, QC J0K 3B0, Canada

**Keywords:** V_2_O_5_/SnO_2_ nanostructures, high specific capacity, energy storage, Li-ion battery

## Abstract

In this study, the facile synthesis of SnO_2_ quantum dot (QD)-garnished V_2_O_5_ nanobelts exhibiting significantly enhanced reversible capacity and outstanding cyclic stability for Li^+^ storage was achieved. Electrochemical impedance analysis revealed strong charge transfer kinetics related to that of V_2_O_5_ nanobelts. The SnO_2_ QD-garnished V_2_O_5_ nanobelts exhibited the highest discharge capacity of ca. 760 mAhg^−1^ at a density of 441 mAg^−1^ between the voltage ranges of 0.0 to 3.0 V, while the pristine V_2_O_5_ nanobelts samples recorded a discharge capacity of ca. 403 mAhg^−1^. The high capacity of QD-garnished nanobelts was achieved as an outcome of their huge surface area of 50.49 m^2^g^−1^ and improved electronic conductivity. Therefore, the as-presented SnO_2_ QD-garnished V_2_O_5_ nanobelts synthesis strategy could produce an ideal material for application in high-performance Li-ion batteries.

## 1. Introduction

The field of electrical energy storage devices has gained popularity owing to their potential for enhanced durability, strong energy performance, and long-term reliability, and they have become a focal point in the renewable energy industry. Due to their huge energy density, low weight, and reliable stability performance, lithium and sodium (SIB) batteries have received a great deal of coverage in the last few decades [1]. As a result of its low ion diffusion and poor capacity of 372 mAhg^−1^, graphite has already exceeded its material capacity as an anode material. As a result, extensive research has been conducted with respect to alloying (M + xLi^+^ + xe^−^ →Li_x_M) [2] and switchover (M_x_O_y_ + 2y Li^+^ + 2y e^−^ →y Li_2_O + xM) [3] reactions, with the aim of synthesizing innovative electrochemical compounds that exhibit a greater tendency for significantly increased Li-ion accommodation.

Transition metal oxides, such as SnO_2_, Fe_3_O_4_, Fe_2_O_3_, V_2_O_5_, TiO_2_, CuO, and Co_3_O_4_, which exhibit electrochemical activity, have recently been established as potential anode electrodes to satisfy the growing demand for high-performance Li-ion batteries (LIB), due to their huge theoretical capacity and relatively extensive availability [4]. For instance, vanadium-based oxide layers, such as V_2_O_5_ (V^5+^), VO_2_ (V^4+^), and V_6_O_13_ (V^3+^), which exhibit multiple oxidation states, have gained significant interest [5]. A large number of synthesis and processing routes have been developed for the application of these materials in LIB fuel cells [6]. In particular, vanadium pentoxide (V_2_O_5_) has significant traction for its potential application as both the cathode [7,8] and anode material in LIBs and SIBs, due to its huge capacity, facile synthesis, high energy density, extensive natural abundance, low price, and large theoretical capacity of 441 mAhg^−1^. The huge theoretical capacity can be accredited to its (V_2_O_5_) capacity to recombine nearly 3Li^+^ ions per mole of V_2_O_5_ + xLi^+^ + xe^−^ ↔ Li_x_V_2_O_5_ and benefits of large energy and high power density, low price, easy production, and improved protection [9]. Pure V_2_O_5_ exhibits a poor ion diffusion coefficient, low electrical conductivity (10^−5^–10^−3^ S cm^−1^), and deprived structural stability, all of which hinder the uptake of V_2_O_5_ as an electrode material in LIBs [10].

SnO_2_ has been studied extensively over the past decade as a potential candidate for the anode material in next-generation LIBs, owing to its high theoretical power of 783 mAhg^−1^ (0.005–1.000 V vs. Li), relative abundance, less price, and environmental friendliness. The conversion reaction occurs when SnO_2_ is reduced to Sn, and an Li_2_O matrix is formed (SnO_2_ + 4Li^+^+ 4e^−^ → Sn + 2Li_2_O, 711 mAhg^−1^). The reversible reaction [Sn + xLi^+^ + xe^−^ ↔ Li_x_Sn (0.0 < x ≤ 4.4), 783 mAhg^−1^] (0.005–1.0 V vs. Li) imparts the reversible potential of the SnO_2_ anode once the first, irreversible, discharge cycle has been completed [4]. The oxides, such as ruthenium oxide, nickel oxide, SnO_2_, In_2_O_3_, Fe_2_O_3_, Co_3_O_4_, and Fe_3_O_4_, can be introduced into the V_2_O_5_ framework, to enhance both its stability and electrical properties, which, in turn, increases the intercalation rate, cycling efficiency, and specific capacity of LIBs.

Liu et al. [11] stated double-shelled V_2_O_5_-SnO_2_-based nanocapsules exhibiting a rescindable potential of 600 mAhg^−1^ and a rate of 250 mAg^−1^ after 50 cycles. Yang et al. [12] used the Ostwald ripening method to produce hollow SnO_2_ nanospheres by handling SnCl_4_ in a C_12_H_22_O_11_ solution under hydrothermal process. After 20 cycles at 0.1 Ag^−1^, the hollow SnO_2_ nanospheres exhibited an ion storage capacity of 520 mAhg^−1^. Sun et al. [13] used an atomic layer deposition approach to deposit a graphene coating on amorphous V_2_O_5_ to improve its electrical conductivity and electrochemical behavior. Feng et al. [14] reported a novel MoO_2_-SnO_2_-C nanocomposite, based on the physical characteristics of the metal-oxide MoO_x_, which exhibited a nontypical performance at high potential, alongside a large specific capacitance and initial Coulombic efficiency (ICE) [15]. The morphology of the MoO_2_-SnO_2_-C nanocomposite resulted in an increase in the concentration of active lithium storage sites, imparting an increased reversible capability and ICE. Du et al. [16] used H_2_ as a decreasing agent to synthesize 80V_2_O_5_-20P_2_O_5_ glass, which exhibited a maximum potential of 243 mAhg^−1^ (0.1 C) after 100 cycles. Yu et al. [17] reported that, as the vanadium content of the glass electrode material increased, so did its electrical conductivity. Jingwei et al. [18], reported on the structural and electrical properties of glass-ceramics in the context of their utilization as a cathode material in LIBs. During their research the proportion of V^3+^ and V^4+^ in the glass-ceramic was steadily increased, and a corresponding increase in the electrical conductivity and specific power was reported.

Herein, SnO_2_ QDs distributed along V_2_O_5_ nanobelts were used as a hybrid, huge-capacity, and storage material in Li-ion batteries. Enhanced electrochemical properties, such as high specific discharge ability and cycling stability, were observed.

## 2. Materials and Methods

### 2.1. V_2_O_5_ Nanobelt Synthesis

V_2_O_5_ nanobelt (vanadium(V) oxide: v NB) synthesis was achieved through the following steps. First, 1 g of commercially available V_2_O_5_ powder was distributed in 60 mL of pure water and stirred for 30 min to impart a uniform particle dispersion. Then, 2 M sodium chloride was dispensed to the aforesaid solution and stirred constantly for 3 days at room temperature. The obtained nanostructures were extracted using centrifugation and cleaned numerous times with pure water and ethanol, before being dried overnight in a vacuum oven at 85 °C.

### 2.2. SnO_2_ QD Garnished V_2_O_5_ Nanobelts Synthesis

Once dried, 0.07 g of the as-synthesized V_2_O_5_ nanobelts were poured to 60 mL of water and stirred for 120 min at 25 °C to obtain a widely dispersed homogeneous solution. Following this, 0.03 g of the SnO_2_ QDs (tin(IV) oxide) were poured to the aforesaid solution and stirred for 180 min at 25 °C. Finally, the products were separated via centrifugation and cleaned using a mixture of water and ethanol between five and seven, having earlier been dried overnight in a vacuum oven at 85 °C.

### 2.3. Preparation of Electrodes

Electrodes were prepared to examine the ability of the synthesized nanostructures in the context of their application in batteries. The electrodes were prepared with an active material, activated carbon (Daejung Chemicals and Metals Co., Siheung-si, Korea), and polyvinylidene fluoride (PVDF, Sigma-Aldrich, Seoul, Korea) weight ratio of 7:2:1. N-methyl-2-pyrrolidone (NMP, purity: >99.5%, Daejung Chemicals and Metals Co., Siheung-si, Korea) as a solvent was added to the constituent materials before mixing uniformly for 30 min using a pestle and mortar. The homogeneous solution was then drop-cast onto pre-cleaned 15-mm-diameter Cu disks at 80 °C and then dried overnight in a vacuum oven at 130 °C.

### 2.4. Characterization

The phase composition of the synthesized nanostructures was determined using X-ray diffraction (XRD; PANalytical X’pert PRO, Etten Leur, The Netherlands), utilizing CuKα radiation. The V NB and SnO_2_ QD-garnished on the V_2_O_5_ nanobelts (vs-73) morphologies were analyzed using scanning electron microscopy (SEM, Hitachi S-4800, Saitama, Japan) and high-resolution transmission electron microscopy (HR-TEM, G2 F30 S-Twin, Seoul, Korea). The electronic structure and chemical states of the samples were investigated using X-ray photoelectron spectroscopy (XPS, Thermo Fisher Scientific MultiLab 2000, Seoul, Korea). Finally, the surface area of each sample was determined using 3Flex (Micromeritics, Norcross, GA, USA).

### 2.5. Electrochemical Analysis

Electrodes were prepared in a coin-cell-battery configuration and were fabricated inside a glove box with CR2032. Each cell formed a Li circular disc with a diameter and thickness of 16 and 0.6 mm, respectively. An electrolyte medium was prepared as follows, 1 M lithium hexafluorophosphate was dissolved in C_3_H_4_O_3_, C_3_H_6_O_3_, and C_5_H_10_O_3_ in a 1:1:1 volume ratio (99%, Sigma-Aldrich, Seoul, Korea) to produce 100 μL of electrolyte. A Celgard disk (Celgard, LLC Corp., Charlotte, NC, USA), with 20 mm diameter and 20 μm thickness, was utilized as the separator. The electrochemical properties of the synthesized v and vs-73 samples were measured in CR2032 coin-type cells using BCS-805 and SP-200 (Bio-Logic, Seyssinet-Pariset, France). To obtain a measure of the synthesized electrode performance, the coin cells were charged/discharged in a voltage window among 0.001 and 3.000 V under varying densities using BCS-805. Cyclic voltammetry (CV) analysis was performed using an SP-200 (Bio-Logic, Seyssinet-Pariset, France) electrochemical workstation at a scan rate of 0.1 mVs^−1^. Electrochemical impedance spectroscopy (EIS) analysis was achieved on the prepared electrode in the applied frequency range among 100 mHz and 200 kHz at an amplitude of 10 mV using an SP-200 (Bio-Logic, Seyssinet-Pariset, France) electrochemical workstation.

## 3. Results and Discussion

**Figure 1:**
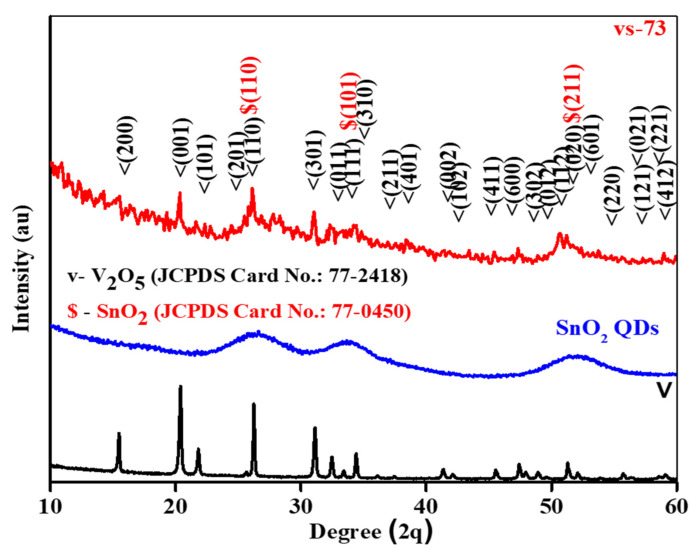
XRD analysis of V_2_O_5_ and SnO_2_ QDs-garnished V_2_O_5_ nanobelts.

XRD was utilized to determine the crystalline structure of both the V_2_O_5_ nanobelts (v NB) and SnO_2_ QD-garnished V_2_O_5_ nanobelts (vs-73), as shown in Figure 1. In the pristine sample, several characteristic (hkl) peaks were observed which corresponded to an orthorhombic crystal structure exhibiting a Pmmn space group as per JCPDS card No. 77-2418. No impurity phases, such as VO, VO_2_, V_4_O_9_, V_3_O_7_, V_6_O_13_, and V_8_O_15_, were detected, confirming the quality of the synthesized sample. In addition, the SnO_2_ QDs-garnished V_2_O_5_ nanobelts exhibited a mixed crystalline structure of the nanobelts and SnO_2_ QDs, suggesting that the samples interacted favorably with one another. Characteristic (hkl) peaks were observed in the SnO_2_ QDs at 26.0° (110), 34.5° (101), and 52.2° (211), which corresponded with a tetragonal crystal structure as per JCPDS card No. 77-0450. It was found that the orthorhombic crystal structure of the V_2_O_5_ nanobelts did not change as a result of the introduction of the SnO_2_ QDs, suggesting that the composition of the QDs was not influenced by the crystal structure of the V_2_O_5_ nanobelts during the deposition process. In addition, SnO_2_ QDs garnished on the surface of V_2_O_5_ nanobelts due to this the X-rays initially exposed maximum on the surface of SnO_2_ QDs compared to V_2_O_5_ nanobelts. Hence, it could be the cause of V_2_O_5_ characteristic peak showed lowered intensities in SnO_2_ QDs-garnished V_2_O_5_ nanobelts samples.

**Figure 2:**
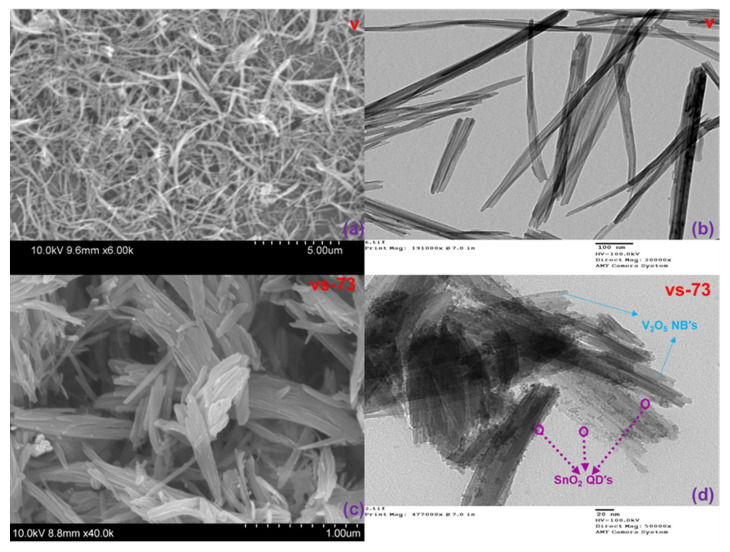
Electron microscopy images: (**a**) SEM image of V_2_O_5_ nanobelts, (**b**) TEM image of V_2_O_5_ nanobelts, (**c**) SEM image of SnO_2_ QDs-garnished V_2_O_5_ nanobelts, and (**d**) TEM image of SnO_2_ QDs-garnished V_2_O_5_ nanobelts.

**Figure 3:**
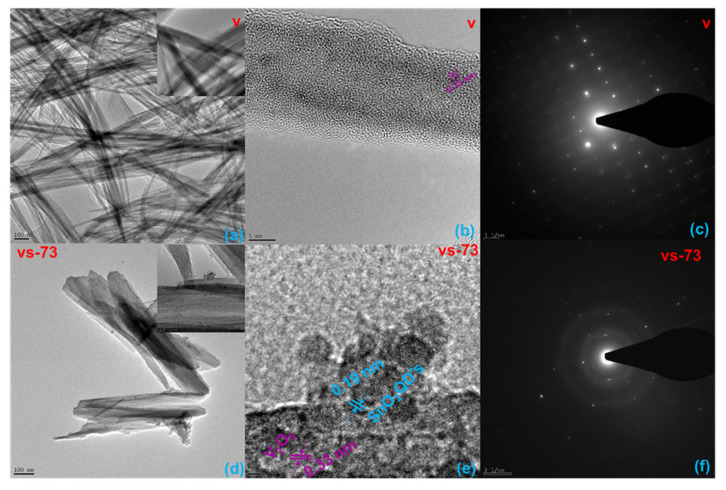
HR-TEM images of (**a**–**c**) V_2_O_5_ nanobelts, and (**d**–**f**) SnO_2_ QDs-garnished V_2_O_5_ nanobelts.

SEM and TEM images of the pristine V_2_O_5_ and the SnO_2_ QD-garnished V_2_O_5_ samples are revealed in Figure 2a–d. Figure 2a displays a SEM image of the pristine V_2_O_5_ sample, confirming the presence of nanobelts of varying morphologies. Figure 2c shows SEM images of the SnO_2_ QD-garnished V_2_O_5_ samples, which confirm the result suggested through XRD analysis that the deposition of SnO_2_ QDs on the V_2_O_5_ nanobelts does not affect its morphology. The TEM image in Figure 2d confirms that the SnO_2_ QDs were uniformly dispersed across the surface of the nanobelts. In addition, the HR-TEM images, lattice fringe patterns, and selected area electron diffraction (SAED) patterns for all prepared materials are given in Figure 3a–f. Figure 3a–c show the morphology, fringe pattern, and SAED patterns of nanobelts exhibiting various morphologies. A lattice fringe width of 0.33 nm, combined with the corresponding SAED pattern, indicates that the synthesized samples adopt a crystalline structure. HR-TEM images of the SnO_2_ QD-garnished V_2_O_5_ nanobelts are shown in Figure 3d–f and depict the SnO_2_ QD distribution across the surface of NBs, with a corresponding lattice fringe width of 0.19 nm. In addition, a strong interconnection is exhibited between the NB and QD samples, which is shown in Figure 3e. The SAED pattern obtained from the SnO_2_ QD-garnished V_2_O_5_ nanobelts exhibited a combination of ring and dot patterns, while the d-value obtained from HR-TEM significantly corresponded with that obtained through XRD analysis.

**Figure 4:**
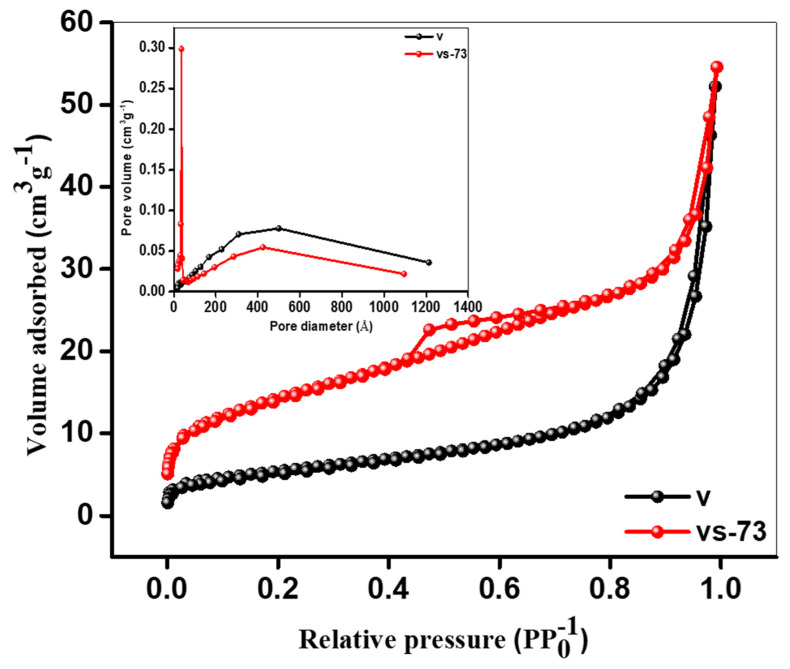
BET analysis of V_2_O_5_ nanobelts, and SnO_2_ QDs-garnished V_2_O_5_ nanobelts.

The surface area of each structure was evaluated using Brunauer–Emmett–Teller (BET) analysis, as revealed in Figure 4. The surface areas of the pristine nanobelts and the SnO_2_ QD-garnished V_2_O_5_ nanobelts were 19.27 and 50.49 m^2^g^−1^, respectively. This significant increase in the surface area suggests that the QDs are successfully distributed along the NBs, with this agglomeration imparting the marked increase in the surface area between samples. This increase in the surface area enhances a greater number of surface active sites, while the volume of electrolyte in interaction with the material also increases and the Li-ion pathway length is reduced.

**Figure 5:**
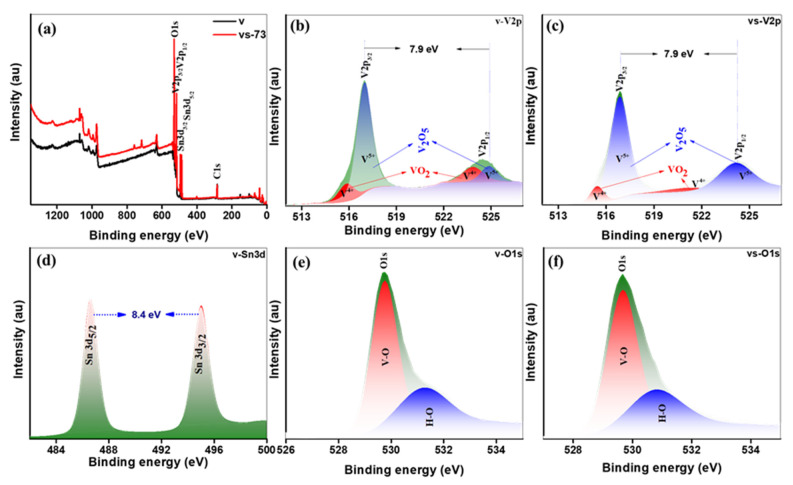
XPS analysis of pristine and SnO_2_ QDs-garnished V_2_O_5_ nanobelts (**a**) survey spectra, (**b**,**c**) V (2p) for pristine and SnO_2_ QDs-garnished V_2_O_5_ nanobelts, (**d**) Sn (3d) for SnO_2_ QDs-garnished V_2_O_5_ nanobelts, and (**e**,**f**) O (1s) for pristine and SnO_2_ QDs-garnished V_2_O_5_ nanobelts.

The chemical states of both the pure V_2_O_5_ nanobelts and SnO_2_ QD-garnished V_2_O_5_ nanobelts were examined using XPS, as presented in Figure 5a–f. Figure 5a displays the survey spectra obtained from each of the samples, which exhibited core peaks corresponding to V (2p), Sn (5d), and O (1s), thus confirming the presence of the constituent elements expected from the molecular formula of each sample. The core-level peaks of the V (2p) spectra gathered from the V_2_O_5_ nanobelts and SnO_2_ QD-garnished V_2_O_5_ nanobelts are revealed in Figure 5b,c. Two significant peaks are observed at energies of 516.7 and 524.8 eV, which related to the V 2p_3/2_ and V 2p_1/2_ energy states in V_2_O_5_, respectively. Both the V 2p_3/2_ and V p_1/2_ peaks were deconvoluted to form two sets of distinct peaks at energies of 515.9 and 516.9 eV and 523.8 and 524.9 eV, respectively. Each set of peaks corresponded to the V^4+^ and V^5+^ oxidation states within the V_2_O_5_ sample, respectively. Furthermore, the energy disparity between the two sets of peaks was 7.9 eV, which confirmed the existence of the V^5+^ oxidation state in the samples. The area occupied by the peaks corresponding to the V^4+^and V^5+^ proposes the presence of oxygen vacancies in the nanobelt lattice to recompense for the low valency V^4+^ ions [19]. Figure 5d shows the core-level binding energy peak of Sn (3d) in the SnO_2_ QD-garnished V_2_O_5_ nanobelts. The core-level Sn (3d) spectrum contained two distinct peaks at energies of 487 and 495 eV, relates to the Sn (3d_5/2_) and Sn (3d_3/2_) energy states of SnO_2_, respectively. The energy disparity among the two peaks was 8.4 eV, which confirmed the existence of the Sn^4+^ oxidation state in the SnO_2_ QD-garnished V_2_O_5_ nanobelts. The binding energy values obtained from these spectra matched with those previously reported in the literature [20]. O (1s) spectra obtained from the V_2_O_5_ nanobelts and SnO_2_ QD–garnished V_2_O_5_ nanobelts are presented in Figure 5e,f. The spectrum was deconvoluted to form two distinct peaks at energies of 529.8 and 531.2 eV in each sample. The peaks observed at 529.8 and 531.2 eV were attributed to the lattice oxygen and absorbed surface oxygen. For comparison, the original and the fitted XPS are shown in Figure S1.

**Figure 6:**
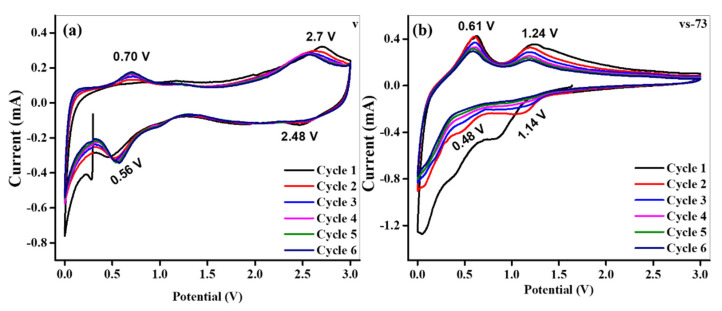
Cyclic voltammograms (CV) performed at scan rate of 1 mV/sec (**a**) pristine, and (**b**) SnO_2_ QDs-garnished V_2_O_5_ nanobelts.

Electrochemical analysis of the V_2_O_5_ and SnO_2_ QD-garnished V_2_O_5_-based electrodes was performed in a two-electrode system combined with a Li disc as the counter, alongside several reference electrodes. Figure 6a,b show the CV graphs obtained from the first six anodic/cathodic (oxidation/reduction) cycles recorded at a scan rate of 0.1 mVs^−1^ over the voltage range of 0.0–3.0 V (vs Li/Li^+^) for the V_2_O_5_ nanobelts and SnO_2_ QD-garnished V_2_O_5_ nanobelts. Two pairs of well-established peaks were noticed at 0.70 and 2.70 V (vs. Li/Li^+^) during the oxidation process and at 0.56 and 2.48 V (vs. Li/Li^+^) throughout the reduction process. The CV curves were obtained from analysis of the V_2_O_5_ nanobelts and were found to correspond to the Li^+^ insertion/extraction process, as given in Figure 6a. The two pairs of distinct peaks were ascribed to the reversible phase transformations which occurred in the Li^+^ intercalated Li_x_V_2_O_5_ phases as follows; α (x < 0.01), ε (0.35 < x < 0.7), δ (0.9 < x ≤ 1), and γ (1 < x < 2) [21].

In addition, the overlapping CV curves acquired between the second and sixth scans reveal strong reversibility cyclability and structural stability. The CV analysis of the SnO_2_ QD-garnished V_2_O_5_ nanobelts is displayed in Figure 6b. During the first reduction scan, the board peak observed at 0.84 V relates to the reaction of SnO_2_ QDs with Li^+^ (SnO_2_ + 4Li^+^ + 4e^−^ → Sn + Li_2_O), which is an irreversible reaction that results in a significant loss of capacity. This process is associated with the creation of a solid electrolyte-interphase (SEI) film on the surface of the electrode, as it is no longer detected in subsequent cycles. SEI film formation often occurs exclusively during the first cycle [22]. In contrast, upon constant Li^+^ insertion, Sn can respond with Li^+^ to procedure Li_x_Sn (0 ≤ x ≤ 4.4) alloys, near to 0.0 V (Sn + xLi^+^ + xe^−^ ↔ Li_x_Sn). In addition, the peaks observed at 0.48 and 1.14 V in the SnO_2_ QD-garnished V_2_O_5_ nanobelt electrodes suggest that the insertion of Li^+^ occurred as a multi-step process. During the subsequent oxidation processes, the sharp robust peak observed at 0.61 V and the wide peak observed at 1.24 V were ascribed to the Li^+^ extraction procedure from Li_x_Sn and the reversible formation of V_2_O_5_, respectively. This result suggests that they share a common reaction pathway. In addition, the oxidation/reduction peaks observed in the SnO_2_ QDs-garnished V_2_O_5_ nanobelts exhibited a marginal shift in comparison to pure V_2_O_5_ nanobelts owing to the response of SnO_2_ with Li^+^ in the Sn metal, a similar phenomenon was reported by Wang et al. [23].

**Figure 7:**
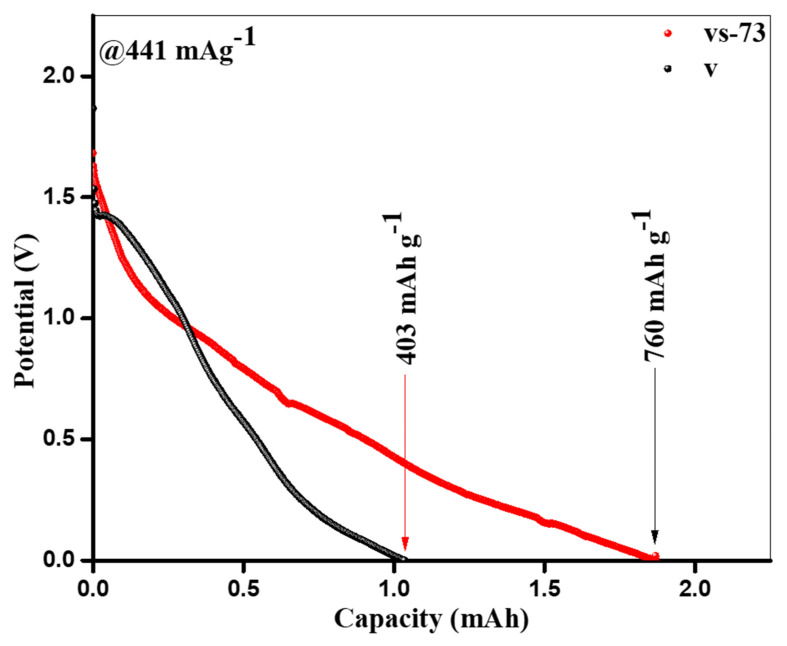
First discharge curves of V_2_O_5_ and SnO_2_ QDs-garnished V_2_O_5_ nanobelts.

**Figure 8:**
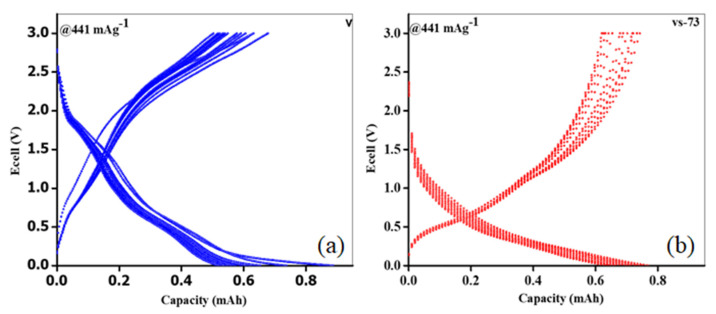
Typical charge/discharge curves of V_2_O_5_ (**a**) and SnO_2_ QDs-garnished V_2_O_5_ nanobelts (**b**) recorded at 441 mAg^−1^.

Figure 7 shows the initial discharge curves obtained from the pure V_2_O_5_ nanobelts and SnO_2_ QD-garnished V_2_O_5_ nanobelts, which exhibited capacities of ca. 403 and 760 mAhg^−1^, respectively, at an applied density of 441 mAg^−1^. During the first discharge cycle, the capacity of the SnO_2_ QD-garnished V_2_O_5_ nanobelts was nearly two times higher than that recorded in the pure V_2_O_5_ nanobelts. This was accredited to the development of a SEI layer on the vs-73 electrode surface. Owing to a number of redox reactions related to Li extraction, several potential plateaus at approximately 0.6, 1.22, and 1.43 V were observed during the initial discharge curves obtained from pure V_2_O_5_ nanobelts and SnO_2_ QDs-garnished V_2_O_5_ nanobelts (Figure 7). Although the theoretical capacity of SnO_2_ is 783 mAhg^−1^ (0.005–1.000–V vs. Li), the storage capacity exhibited by the Li^+^ ions in the SnO_2_ QDs-garnished V_2_O_5_ nanobelts originates exclusively from the V_2_O_5_ nanobelts as only 30 mg SnO_2_ QDs are distributed across its’ surface. As the vs-73 sample is discharged to 0.0 V (vs. Li/Li^+^), V_2_O_5_ exhibits a simultaneous full reduction to form metallic V [24]. However, the capacity of the SnO_2_ QD-garnished V_2_O_5_ nanobelts is shown to be greater than the theoretical capacity of the bulk material (441 mAhg^−1^). It is possible that the activated carbon black used in the electrode may store a limited number of lithium atoms. However, its capacity to do so is minimal. Therefore, it is likely that the significant volume of surface defects, alongside the high surface-area-to-volume ratio exhibited by SnO_2_ QD-garnished V_2_O_5_ nanobelts, effectively increased its Li storage capacity. Typical successive cycles for pure V_2_O_5_ nanobelts and SnO_2_ QD-garnished V_2_O_5_ nanobelts, upon discharge at a density of 441 mAg^−1^, are presented in Figure 8a,b. In pure V_2_O_5_ nanobelts, multiple plateaus observed at ~0.7 and 1.9 V may be ascribed to the reduction in valency of V^+5^ to V^+4^, and V^+4^ to V^+3^, respectively. These plateaus are observed in the pure nanobelts across all current densities, however, this data is not shown. In contrast, these plateaus were not observed in the SnO_2_ QD-garnished V_2_O_5_ nanobelts, and, instead, a steady decrease in potential was recorded across the various current densities. A typical charge/discharge graph obtained at 441 mAg^−1^ is revealed in Figure 8b.

**Figure 9:**
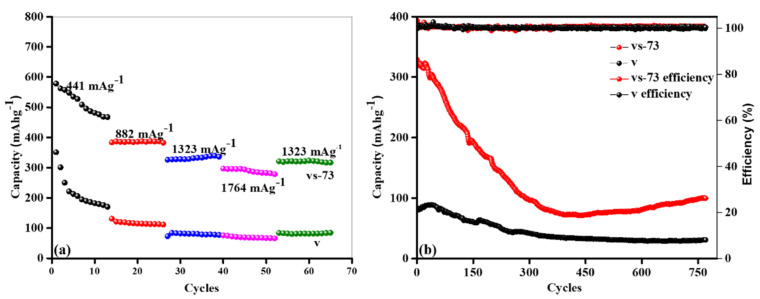
Pristine and SnO_2_ QDs-garnished V_2_O_5_ nanobelts: (**a**) capacity at various current densities; (**b**) capacity vs. cycle number plots: stability test and efficiency of the cell.

The capacities of both the pure V_2_O_5_ nanobelts and the SnO_2_ QDs-garnished V_2_O_5_ nanobelts were estimated at several applied densities among 441 and 1323 mAg^−1^ across a voltage window of 0.001 to 3.000 V, and the results are shown in Figure 9a. The SnO_2_ QDs-garnished V_2_O_5_ nanobelts exhibited a minor reduction in the storage capacity as the density enhanced. For instance, at a large applied current density of 1764 mAg^−1^, the vs-73 sample was able to deliver a comparatively huge, stable, rescindable capacity of ~296.6 mAhg^−1^. Unusually, when the applied current density was applied back to 1323 mAg^−1^ after 10 cycles, an excellent rescindable storage capacity of ~321.1 mAhg^−1^ was obtained, thus proving the extraordinary rate capability exhibited by the vs-73 sample. However, the pure V_2_O_5_ nanobelts exhibited a rapid decrease in current density when compared to that of the SnO_2_ QDs-garnished V_2_O_5_ nanobelts. At an increased current density of 1764 mAg^−1^, a capacity of 66.3 mAhg^−1^ was recorded, which is approximately 4.5 times lower than that exhibited by the SnO_2_ QDs-garnished V_2_O_5_ nanobelts. These results suggest that even a small amount of SnO_2_ QDs dispersed across the surface of the V_2_O_5_ nanobelts can drastically improve the capacity of the material, which may be an effect of the induced upsurge in the surface area (shown in BET analysis) and defect concentration. To examine the stability of each cell sample, the pure and SnO_2_ QDs garnished nanobelts were subjected to up to 770 oxidation/reduction cycles, the outcomes of which are revealed in Figure 9b. It was observed that the vs-73 sample exhibited a rapid decrease in its capacity after 300 charge/discharge cycles, this was in contrast to the behavior exhibited by the pure nanobelts, which exhibited a constant capacity for the duration of the test. However, the SnO_2_ QD-garnished V_2_O_5_ nanobelts exhibited an overall capacity 3.2 times that exhibited by pure nanobelts, while retaining a Coulombic efficiency of ~100% after 770 cycles.

**Figure 10:**
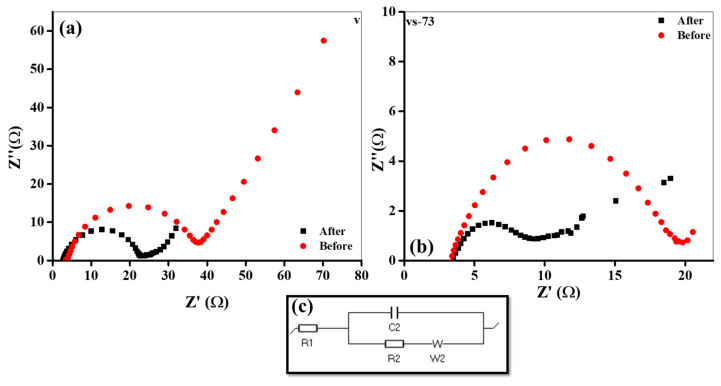
(**a**,**b**) Nyquist plots (Z′ vs. −Z″) EIS of before and after cycling, and (**c**) fitted equivalent circuit.

Electrochemical impedance spectroscopy (EIS) was recorded to determine the kinetics of the synthesized electrodes, as shown in Figure 10a–c. Figure 10a,b show the Nyquist plots (Z vs. −Z′) recorded before and after the cycles performed in both pristine nanobelts and SnO_2_ QD-garnished V_2_O_5_ nanobelts. The impedance spectra were fitted using a simplified physical equivalent circuit, as revealed in Figure 10c. The circuit consists of the electrolyte, the charge transfer resistance, the double-layer capacitance, and the Warburg impedance; which were denoted by R1, R2, C2, and W2, respectively. The Nyquist plots obtained from the pristine nanobelts and the SnO_2_ QD-garnished V_2_O_5_ nanobelts contain an arc in the applied high-frequency region and an inclined straight line in the low-frequency region. The R1 value was significantly reduced from 4 to 3 Ω, indicating that the electrolyte conductivity was improved with the introduction of QDs. The semicircle observed in the high-frequency region pertains to a charge transfer resistance (R2). The radius of the semicircle recorded in the vs-73 sample was significantly lesser than that of the V_2_O_5_ sample, which represented a faster charge transfer in the vs-73 electrode. The R2 values calculated before and after each cycle, were obtained through an estimation of the radius of the arc observed at high-frequencies. The resulting values were 14 and 5 Ω, and 28 and 19 Ω for the NBs and the SnO_2_ QD-garnished V_2_O_5_ nanobelts, respectively. The smaller R2 values observed in the pristine NBs confirmed the increased charge transfer in the SnO_2_ QDs-garnished V_2_O_5_ nanobelts as a result of the introduction of SnO_2_ QDs across the surface of the nanobelts. The significant decrease in the R2 values were ascribed to the large surface area of the distinctively hierarchical structure of the material, alongside the strong electronic conductivity exhibited by the synthesized nanostructures. The large surface area exhibited by the samples increased the active interaction area among the solid-liquid interfaces and shortened the ion diffusion path, imparting an increased ion diffusion rate alongside improved charge transfer. Similarly, the presence of oxygen vacancies, as validated through XPS analysis, may speed up the reaction rate as they may act as potential nucleation points in the Li^+^ ion charge/discharge process (a process that has been previously reported in literature) [25].

## 4. Conclusions

In this study, pristine V_2_O_5_ nanobelts and SnO_2_ QD-garnished V_2_O_5_ nanobelts were successfully synthesized via a facile method for energy storage applications. The vs-73 sample exhibited a surface area of 50 m^2^g^−1^, which is 2.6 times that of the pure V_2_O_5_ nanobelts at 19 m^2^g^−1^. A significant improvement in the charge transfer kinetics was observed in the SnO_2_ QD-garnished V_2_O_5_ nanobelts in comparison to the pristine V_2_O_5_ nanobelts. The largest discharge capacity of approximately 300 mAhg^−1^ was observed in the vs-73 sample at an applied current density of 1764 mAg^−1^. This value was retained over 770 cycles, alongside a Coulombic efficiency of ~100%, suggesting that this material exhibits discharge and stability values that make it an ideal candidate for energy storage applications.

## Data Availability

Not applicable.

## Supplementary Materials

The following are available online, Figure S1: Deconvolution of XPS analysis for all the synthesized samples.

## Sample Availability

Samples of the compounds are not available from the authors.
