# Supplementary material for: SnO2 Quantum Dots Distributed along V2O5 Nanobelts for Utilization as a High-Capacity Storage Hybrid Material in Li-Ion Batteries
Source: Molecules. 2021 Nov 30;26(23):7262. doi: 10.3390/molecules26237262 (PMC8658980; doi:10.3390/molecules26237262)
Supplement: Supplementary file 1 [file molecules-26-07262-s001.zip › molecules-1494041-supplementary.pdf]

Supplementary Materials

SnO_2 Quantum Dots Distributed Along V_2O_5 Nanobelts for Utilization As a High-Capacity Storage Hybrid Material in Li-ion Batteries

I. Neelakanta Reddy [1,*,†], Bhargav Akkinepally [1,†], V. Manjunath [2], G. Neelima [3], M. V. Reddy [4,*] and Jaesool Shim [1,*]

[1] School of Mechanical Engineering, Yeungnam University, Gyeongsan 38541, Korea; bhargav.aero@gmail.com
[2] Department of Physics, Sri Padmavati Mahila Visvavidyalayam, Tirupati 517502, India; drvmanju18@gmail.com
[3] Department of Physics, National Taiwan University, Taipei 10617, Taiwan; neelimareddy2011@gmail.com
[4] Nouveau Monde Graphite, Saint Michel Des saints, Québec, JOk380, Canada
* Correspondence: neela.sra@gmail.com (I.N. R.); reddymvvr@gmail.com (M.V.R.); jshim@ynu.ac.kr (J.S.)
† Equally contributed first author.

Keywords: V_2O_5 /SnO_2 nanostructures; high specific capacity; energy storage; Li-ion battery

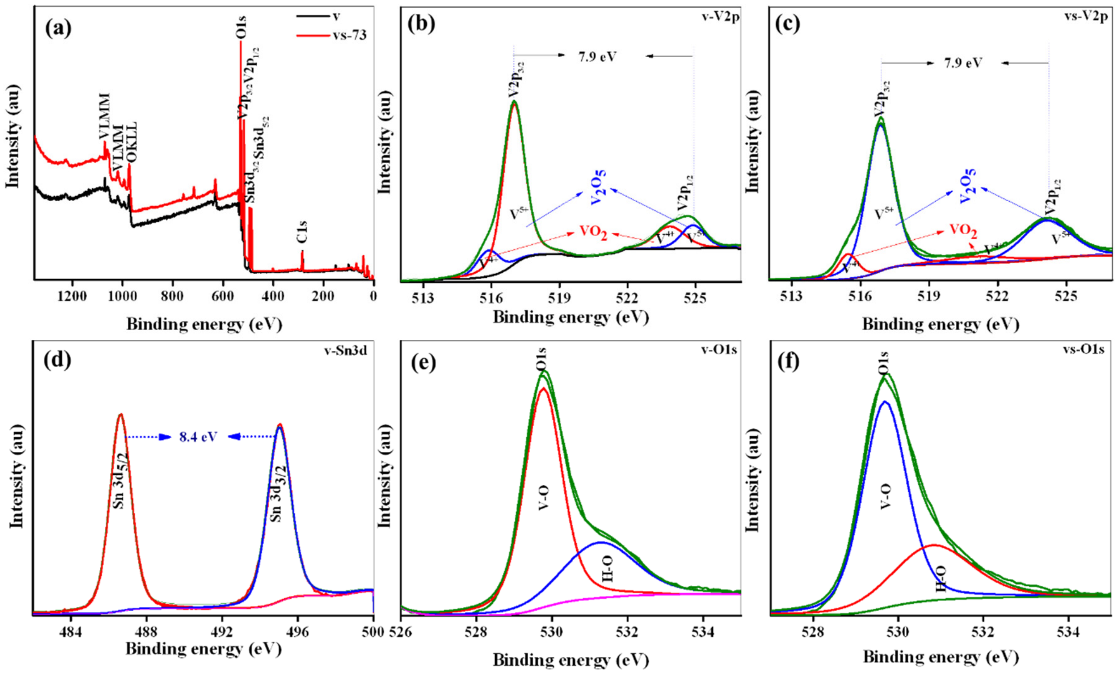

Figure. S1: Deconvolution of XPS analysis for all the synthesized samples.
